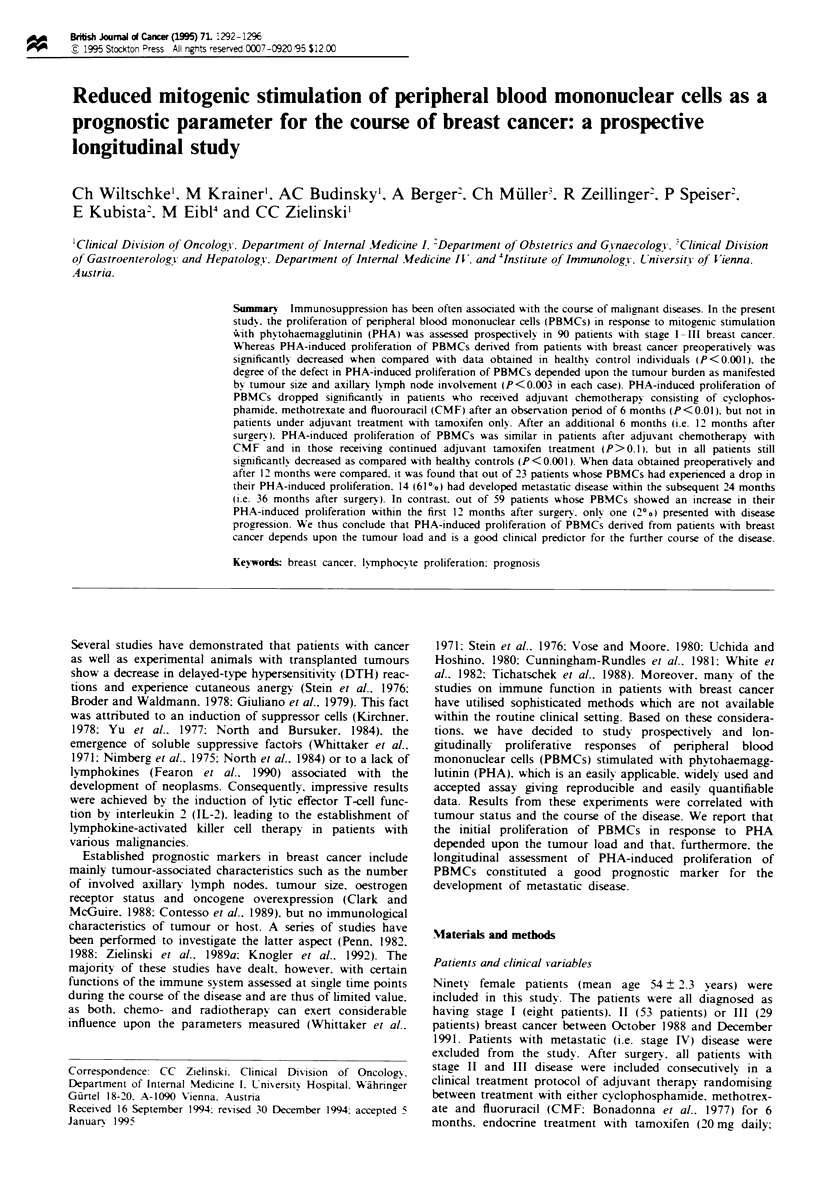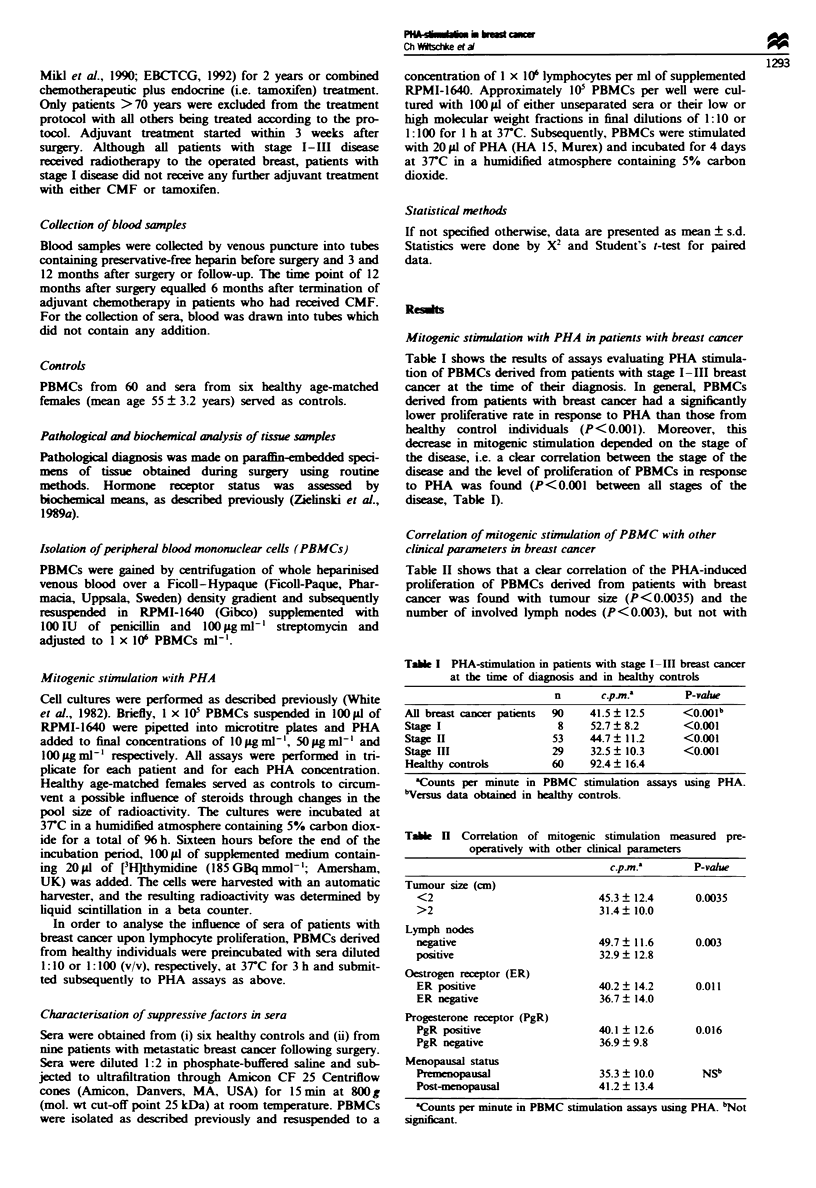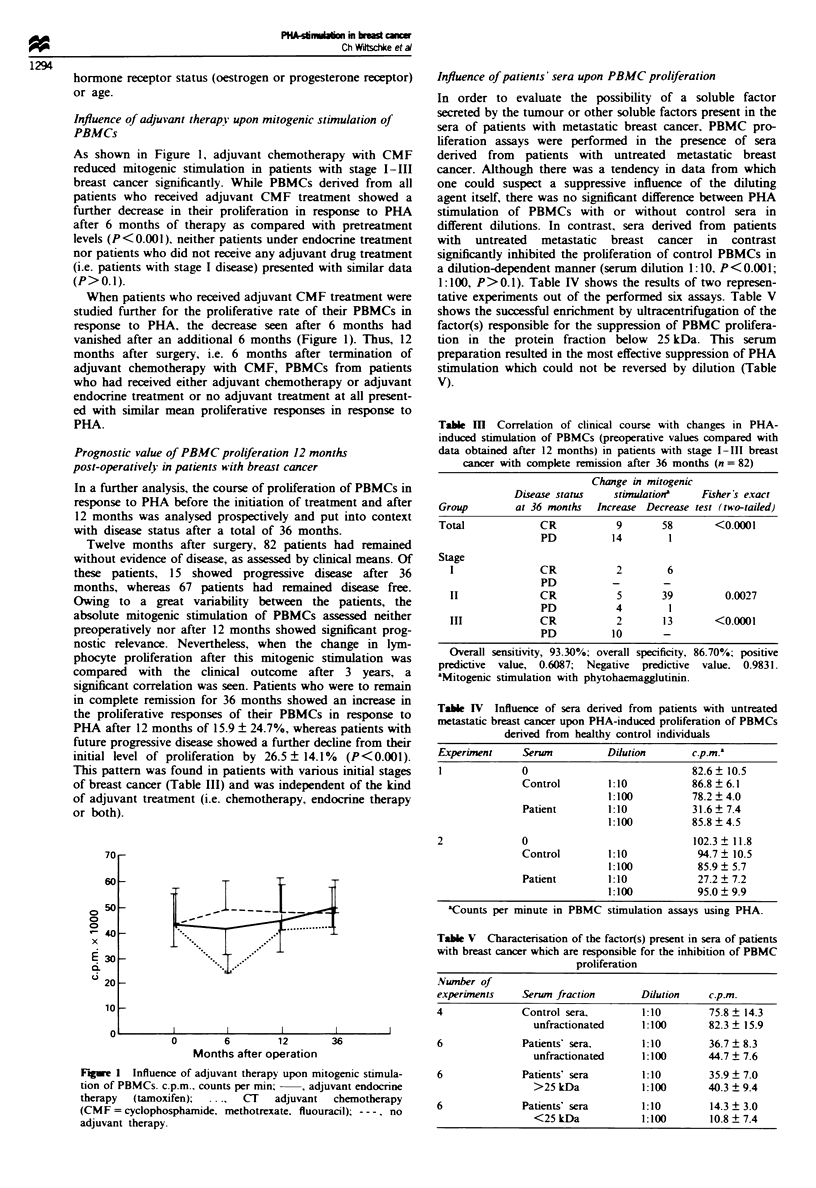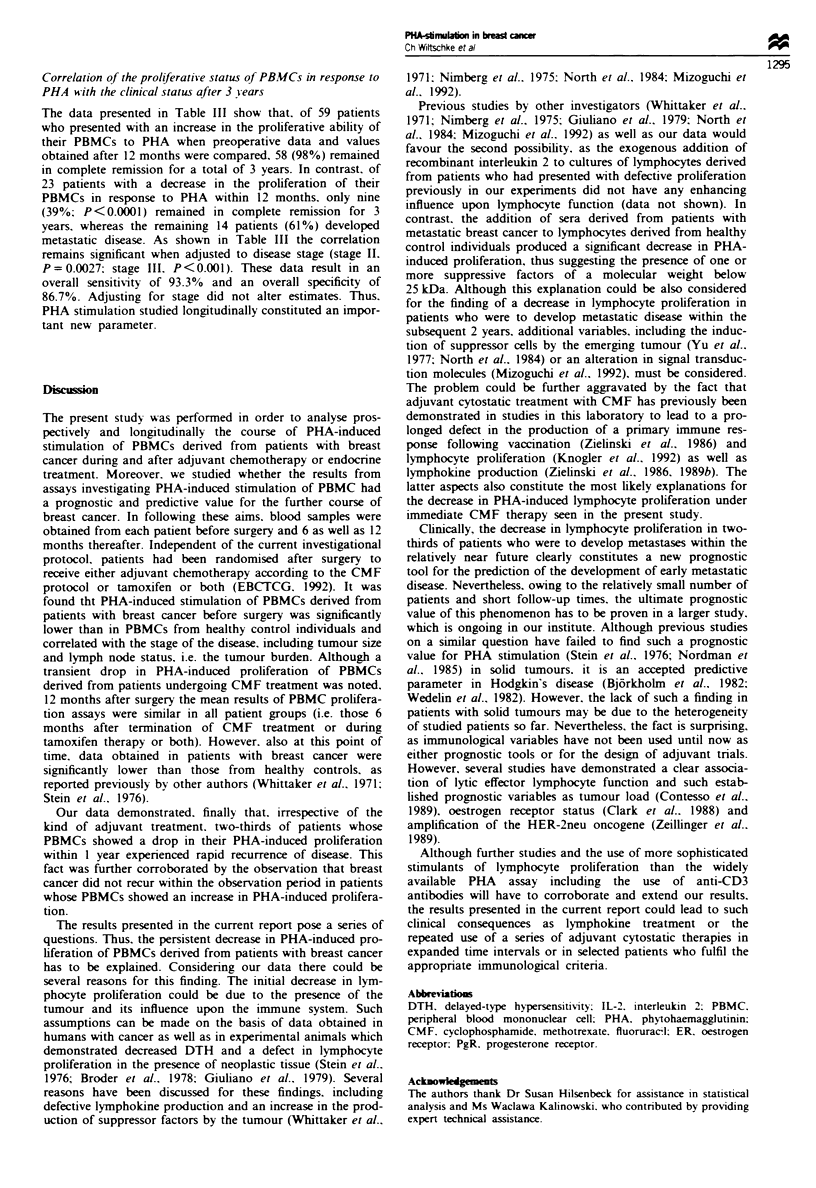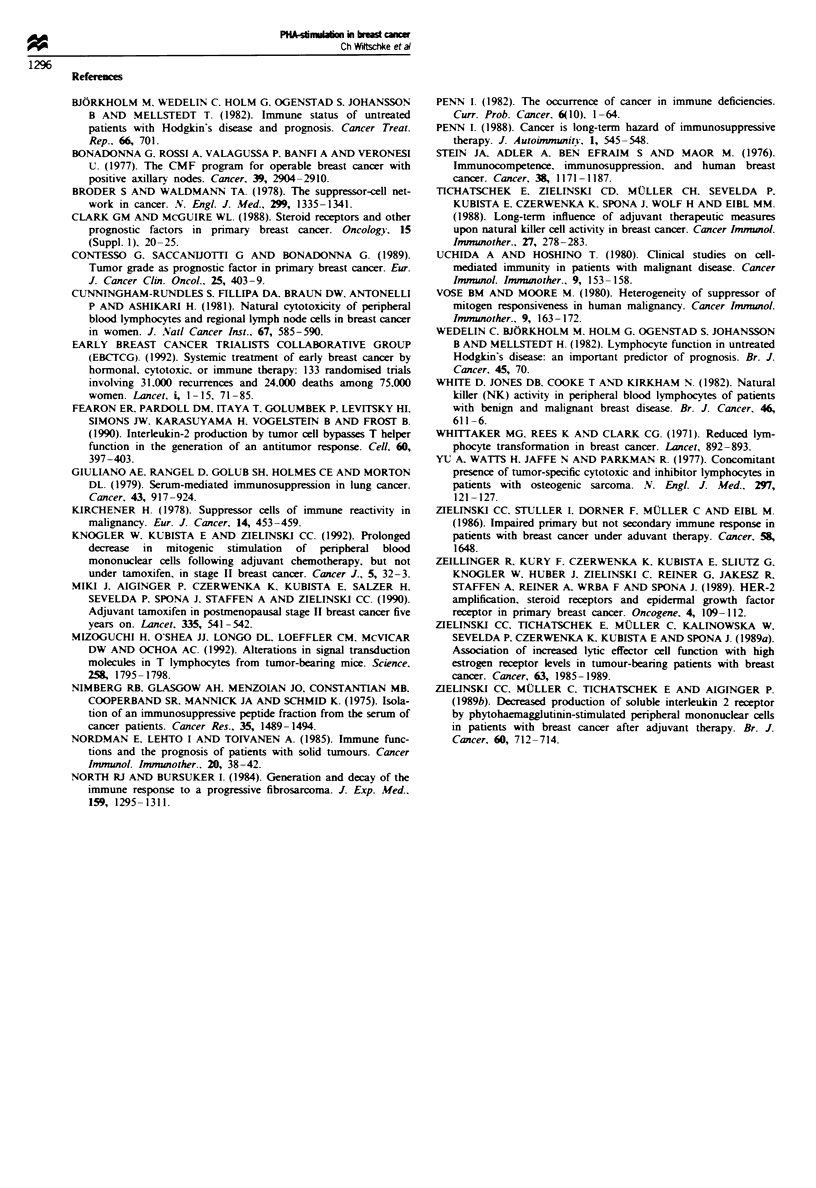# Reduced mitogenic stimulation of peripheral blood mononuclear cells as a prognostic parameter for the course of breast cancer: a prospective longitudinal study.

**DOI:** 10.1038/bjc.1995.250

**Published:** 1995-06

**Authors:** C. Wiltschke, M. Krainer, A. C. Budinsky, A. Berger, C. Müller, R. Zeillinger, P. Speiser, E. Kubista, M. Eibl, C. C. Zielinski

**Affiliations:** Department of Internal Medicine, University of Vienna, Austria.

## Abstract

Immunosuppression has been often associated with the course of malignant diseases. In the present study, the proliferation of peripheral blood mononuclear cells (PBMCs) in response to mitogenic stimulation with phytohaemagglutinin (PHA) was assessed prospectively in 90 patients with stage I-III breast cancer. Whereas PHA-induced proliferation of PBMCs derived from patients with breast cancer preoperatively was significantly decreased when compared with data obtained in healthy control individuals (P < 0.001), the degree of the defect in PHA-induced proliferation of PBMCs depended upon the tumour burden as manifested by tumour size and axillary lymph node involvement (P < 0.003 in each case). PHA-induced proliferation of PBMCs dropped significantly in patients who received adjuvant chemotherapy consisting of cyclophosphamide, methotrexate and fluorouracil (CMF) after an observation period of 6 months (P < 0.01), but not in patients under adjuvant treatment with tamoxifen only. After an additional 6 months (i.e. 12 months after surgery), PHA-induced proliferation of PBMCs was similar in patients after adjuvant chemotherapy with CMF and in those receiving continued adjuvant tamoxifen treatment (P > 0.1), but in all patients still significantly decreased as compared with healthy controls (P < 0.001). When data obtained preoperatively and after 12 months were compared, it was found that out of 23 patients whose PBMCs had experienced a drop in their PHA-induced proliferation, 14 (61%) had developed metastatic disease within the subsequent 24 months (i.e. 36 months after surgery). In contrast, out of 59 patients whose PBMCs showed an increase in their PHA-induced proliferation within the first 12 months after surgery, only one (2%) presented with disease progression. We thus conclude that PHA-induced proliferation of PBMCs derived from patients with breast cancer depends upon the tumour load and is a good clinical predictor for the further course of the disease.


British Journal of Cancer (1995) 71, 1292-1296

© 1995 Stockton Press All rights reserved 0007-0920/95 $12.00

### Reduced mitogenic stimulation of peripheral blood mononuclear cells as a prognostic parameter for the course of breast cancer: a prospective longitudinal study

Ch Wiltschke[1], M Krainer[1], AC Budinsky[1], A Berger[2], Ch Müller[3], R Zeillinger[2], P Speiser[2], E Kubista[2], M Eibl[4] and CC Zielinski[1]

[1]*Clinical Division of Oncology, Department of Internal Medicine I, [2]Department of Obstetrics and Gynaecology, [3]Clinical Division of Gastroenterology and Hepatology, Department of Internal Medicine IV, and [4]Institute of Immunology, University of Vienna, Austria.*

**Summary** Immunosuppression has been often associated with the course of malignant diseases. In the present study, the proliferation of peripheral blood mononuclear cells (PBMCs) in response to mitogenic stimulation with phytohaemagglutinin (PHA) was assessed prospectively in 90 patients with stage I–III breast cancer. Whereas PHA-induced proliferation of PBMCs derived from patients with breast cancer preoperatively was significantly decreased when compared with data obtained in healthy control individuals ($P<0.001$), the degree of the defect in PHA-induced proliferation of PBMCs depended upon the tumour burden as manifested by tumour size and axillary lymph node involvement ($P<0.003$ in each case). PHA-induced proliferation of PBMCs dropped significantly in patients who received adjuvant chemotherapy consisting of cyclophosphamide, methotrexate and fluorouracil (CMF) after an observation period of 6 months ($P<0.01$), but not in patients under adjuvant treatment with tamoxifen only. After an additional 6 months (i.e. 12 months after surgery), PHA-induced proliferation of PBMCs was similar in patients after adjuvant chemotherapy with CMF and in those receiving continued adjuvant tamoxifen treatment ($P>0.1$), but in all patients still significantly decreased as compared with healthy controls ($P<0.001$). When data obtained preoperatively and after 12 months were compared, it was found that out of 23 patients whose PBMCs had experienced a drop in their PHA-induced proliferation, 14 (61%) had developed metastatic disease within the subsequent 24 months (i.e. 36 months after surgery). In contrast, out of 59 patients whose PBMCs showed an increase in their PHA-induced proliferation within the first 12 months after surgery, only one (2%) presented with disease progression. We thus conclude that PHA-induced proliferation of PBMCs derived from patients with breast cancer depends upon the tumour load and is a good clinical predictor for the further course of the disease.

**Keywords:** breast cancer, lymphocyte proliferation; prognosis

Several studies have demonstrated that patients with cancer as well as experimental animals with transplanted tumours show a decrease in delayed-type hypersensitivity (DTH) reactions and experience cutaneous anergy (Stein *et al.*, 1976; Broder and Waldmann, 1978; Giuliano *et al.*, 1979). This fact was attributed to an induction of suppressor cells (Kirchner, 1978; Yu *et al.*, 1977; North and Bursuker, 1984), the emergence of soluble suppressive factors (Whittaker *et al.*, 1971; Nimberg *et al.*, 1975; North *et al.*, 1984) or to a lack of lymphokines (Fearon *et al.*, 1990) associated with the development of neoplasms. Consequently, impressive results were achieved by the induction of lytic effector T-cell function by interleukin 2 (IL-2), leading to the establishment of lymphokine-activated killer cell therapy in patients with various malignancies.

Established prognostic markers in breast cancer include mainly tumour-associated characteristics such as the number of involved axillary lymph nodes, tumour size, oestrogen receptor status and oncogene overexpression (Clark and McGuire, 1988; Contesso *et al.*, 1989), but no immunological characteristics of tumour or host. A series of studies have been performed to investigate the latter aspect (Penn, 1982, 1988; Zielinski *et al.*, 1989*a*; Knogler *et al.*, 1992). The majority of these studies have dealt, however, with certain functions of the immune system assessed at single time points during the course of the disease and are thus of limited value, as both, chemo- and radiotherapy can exert considerable influence upon the parameters measured (Whittaker *et al.*, 1971; Stein *et al.*, 1976; Vose and Moore, 1980; Uchida and Hoshino, 1980; Cunningham-Rundles *et al.*, 1981; White *et al.*, 1982; Tichatschek *et al.*, 1988). Moreover, many of the studies on immune function in patients with breast cancer have utilised sophisticated methods which are not available within the routine clinical setting. Based on these considerations, we have decided to study prospectively and longitudinally proliferative responses of peripheral blood mononuclear cells (PBMCs) stimulated with phytohaemagglutinin (PHA), which is an easily applicable, widely used and accepted assay giving reproducible and easily quantifiable data. Results from these experiments were correlated with tumour status and the course of the disease. We report that the initial proliferation of PBMCs in response to PHA depended upon the tumour load and that, furthermore, the longitudinal assessment of PHA-induced proliferation of PBMCs constituted a good prognostic marker for the development of metastatic disease.

Correspondence: CC Zielinski, Clinical Division of Oncology, Department of Internal Medicine I, University Hospital, Währinger Gürtel 18-20, A-1090 Vienna, Austria
Received 16 September 1994; revised 30 December 1994; accepted 5 January 1995

### Materials and methods

### *Patients and clinical variables*

Ninety female patients (mean age 54 ± 2.3 years) were included in this study. The patients were all diagnosed as having stage I (eight patients), II (53 patients) or III (29 patients) breast cancer between October 1988 and December 1991. Patients with metastatic (i.e. stage IV) disease were excluded from the study. After surgery, all patients with stage II and III disease were included consecutively in a clinical treatment protocol of adjuvant therapy randomising between treatment with either cyclophosphamide, methotrexate and fluoruracil (CMF; Bonadonna *et al.*, 1977) for 6 months, endocrine treatment with tamoxifen (20 mg daily;

Mikl *et al.*, 1990; EBCTCG, 1992) for 2 years or combined chemotherapeutic plus endocrine (i.e. tamoxifen) treatment. Only patients >70 years were excluded from the treatment protocol with all others being treated according to the protocol. Adjuvant treatment started within 3 weeks after surgery. Although all patients with stage I–III disease received radiotherapy to the operated breast, patients with stage I disease did not receive any further adjuvant treatment with either CMF or tamoxifen.

*Collection of blood samples*

Blood samples were collected by venous puncture into tubes containing preservative-free heparin before surgery and 3 and 12 months after surgery or follow-up. The time point of 12 months after surgery equalled 6 months after termination of adjuvant chemotherapy in patients who had received CMF. For the collection of sera, blood was drawn into tubes which did not contain any addition.

*Controls*

PBMCs from 60 and sera from six healthy age-matched females (mean age 55 ± 3.2 years) served as controls.

*Pathological and biochemical analysis of tissue samples*

Pathological diagnosis was made on paraffin-embedded specimens of tissue obtained during surgery using routine methods. Hormone receptor status was assessed by biochemical means, as described previously (Zielinski *et al.*, 1989*a*).

*Isolation of peripheral blood mononuclear cells (PBMCs)*

PBMCs were gained by centrifugation of whole heparinised venous blood over a Ficoll–Hypaque (Ficoll-Paque, Pharmacia, Uppsala, Sweden) density gradient and subsequently resuspended in RPMI-1640 (Gibco) supplemented with 100 IU of penicillin and 100 $\mu g\ ml^{-1}$ streptomycin and adjusted to $1 \times 10^6$ PBMCs $ml^{-1}$.

*Mitogenic stimulation with PHA*

Cell cultures were performed as described previously (White *et al.*, 1982). Briefly, $1 \times 10^5$ PBMCs suspended in 100 µl of RPMI-1640 were pipetted into microtitre plates and PHA added to final concentrations of 10 $\mu g\ ml^{-1}$, 50 $\mu g\ ml^{-1}$ and 100 $\mu g\ ml^{-1}$ respectively. All assays were performed in triplicate for each patient and for each PHA concentration. Healthy age-matched females served as controls to circumvent a possible influence of steroids through changes in the pool size of radioactivity. The cultures were incubated at 37°C in a humidified atmosphere containing 5% carbon dioxide for a total of 96 h. Sixteen hours before the end of the incubation period, 100 µl of supplemented medium containing 20 µl of [$^3$H]thymidine (185 GBq $mmol^{-1}$; Amersham, UK) was added. The cells were harvested with an automatic harvester, and the resulting radioactivity was determined by liquid scintillation in a beta counter.

In order to analyse the influence of sera of patients with breast cancer upon lymphocyte proliferation, PBMCs derived from healthy individuals were preincubated with sera diluted 1:10 or 1:100 (v/v), respectively, at 37°C for 3 h and submitted subsequently to PHA assays as above.

*Characterisation of suppressive factors in sera*

Sera were obtained from (i) six healthy controls and (ii) from nine patients with metastatic breast cancer following surgery. Sera were diluted 1:2 in phosphate-buffered saline and subjected to ultrafiltration through Amicon CF 25 Centriflow cones (Amicon, Danvers, MA, USA) for 15 min at 800 *g* (mol. wt cut-off point 25 kDa) at room temperature. PBMCs were isolated as described previously and resuspended to a concentration of $1 \times 10^6$ lymphocytes per ml of supplemented RPMI-1640. Approximately $10^5$ PBMCs per well were cultured with 100 µl of either unseparated sera or their low or high molecular weight fractions in final dilutions of 1:10 or 1:100 for 1 h at 37°C. Subsequently, PBMCs were stimulated with 20 µl of PHA (HA 15, Murex) and incubated for 4 days at 37°C in a humidified atmosphere containing 5% carbon dioxide.

*Statistical methods*

If not specified otherwise, data are presented as mean ± s.d. Statistics were done by $X^2$ and Student's *t*-test for paired data.

### Results

*Mitogenic stimulation with PHA in patients with breast cancer*

Table I shows the results of assays evaluating PHA stimulation of PBMCs derived from patients with stage I–III breast cancer at the time of their diagnosis. In general, PBMCs derived from patients with breast cancer had a significantly lower proliferative rate in response to PHA than those from healthy control individuals ($P<0.001$). Moreover, this decrease in mitogenic stimulation depended on the stage of the disease, i.e. a clear correlation between the stage of the disease and the level of proliferation of PBMCs in response to PHA was found ($P<0.001$ between all stages of the disease, Table I).

*Correlation of mitogenic stimulation of PBMC with other clinical parameters in breast cancer*

Table II shows that a clear correlation of the PHA-induced proliferation of PBMCs derived from patients with breast cancer was found with tumour size ($P<0.0035$) and the number of involved lymph nodes ($P<0.003$), but not with

**Table I** PHA-stimulation in patients with stage I–III breast cancer at the time of diagnosis and in healthy controls

| | n | *c.p.m.*[a] | P-*value* |
|---|---|---|---|
| All breast cancer patients | 90 | 41.5 ± 12.5 | <0.001[b] |
| Stage I | 8 | 52.7 ± 8.2 | <0.001 |
| Stage II | 53 | 44.7 ± 11.2 | <0.001 |
| Stage III | 29 | 32.5 ± 10.3 | <0.001 |
| Healthy controls | 60 | 92.4 ± 16.4 | |

[a]Counts per minute in PBMC stimulation assays using PHA. [b]Versus data obtained in healthy controls.

**Table II** Correlation of mitogenic stimulation measured pre-operatively with other clinical parameters

| | *c.p.m.*[a] | P-*value* |
|---|---|---|
| Tumour size (cm) | | |
| <2 | 45.3 ± 12.4 | 0.0035 |
| >2 | 31.4 ± 10.0 | |
| Lymph nodes | | |
| negative | 49.7 ± 11.6 | 0.003 |
| positive | 32.9 ± 12.8 | |
| Oestrogen receptor (ER) | | |
| ER positive | 40.2 ± 14.2 | 0.011 |
| ER negative | 36.7 ± 14.0 | |
| Progesterone receptor (PgR) | | |
| PgR positive | 40.1 ± 12.6 | 0.016 |
| PgR negative | 36.9 ± 9.8 | |
| Menopausal status | | |
| Premenopausal | 35.3 ± 10.0 | NS[b] |
| Post-menopausal | 41.2 ± 13.4 | |

[a]Counts per minute in PBMC stimulation assays using PHA. [b]Not significant.

hormone receptor status (oestrogen or progesterone receptor) or age.

### *Influence of adjuvant therapy upon mitogenic stimulation of PBMCs*

As shown in Figure 1, adjuvant chemotherapy with CMF reduced mitogenic stimulation in patients with stage I–III breast cancer significantly. While PBMCs derived from all patients who received adjuvant CMF treatment showed a further decrease in their proliferation in response to PHA after 6 months of therapy as compared with pretreatment levels ($P < 0.001$), neither patients under endocrine treatment nor patients who did not receive any adjuvant drug treatment (i.e. patients with stage I disease) presented with similar data ($P > 0.1$).

When patients who received adjuvant CMF treatment were studied further for the proliferative rate of their PBMCs in response to PHA, the decrease seen after 6 months had vanished after an additional 6 months (Figure 1). Thus, 12 months after surgery, i.e. 6 months after termination of adjuvant chemotherapy with CMF, PBMCs from patients who had received either adjuvant chemotherapy or adjuvant endocrine treatment or no adjuvant treatment at all presented with similar mean proliferative responses in response to PHA.

### *Prognostic value of PBMC proliferation 12 months post-operatively in patients with breast cancer*

In a further analysis, the course of proliferation of PBMCs in response to PHA before the initiation of treatment and after 12 months was analysed prospectively and put into context with disease status after a total of 36 months.

Twelve months after surgery, 82 patients had remained without evidence of disease, as assessed by clinical means. Of these patients, 15 showed progressive disease after 36 months, whereas 67 patients had remained disease free. Owing to a great variability between the patients, the absolute mitogenic stimulation of PBMCs assessed neither preoperatively nor after 12 months showed significant prognostic relevance. Nevertheless, when the change in lymphocyte proliferation after this mitogenic stimulation was compared with the clinical outcome after 3 years, a significant correlation was seen. Patients who were to remain in complete remission for 36 months showed an increase in the proliferative responses of their PBMCs in response to PHA after 12 months of 15.9 ± 24.7%, whereas patients with future progressive disease showed a further decline from their initial level of proliferation by 26.5 ± 14.1% ($P < 0.001$). This pattern was found in patients with various initial stages of breast cancer (Table III) and was independent of the kind of adjuvant treatment (i.e. chemotherapy, endocrine therapy or both).

**Figure 1** Influence of adjuvant therapy upon mitogenic stimulation of PBMCs. c.p.m., counts per min; ——, adjuvant endocrine therapy (tamoxifen); ...., CT adjuvant chemotherapy (CMF = cyclophosphamide, methotrexate, fluouracil); - - -, no adjuvant therapy.

### *Influence of patients' sera upon PBMC proliferation*

In order to evaluate the possibility of a soluble factor secreted by the tumour or other soluble factors present in the sera of patients with metastatic breast cancer, PBMC proliferation assays were performed in the presence of sera derived from patients with untreated metastatic breast cancer. Although there was a tendency in data from which one could suspect a suppressive influence of the diluting agent itself, there was no significant difference between PHA stimulation of PBMCs with or without control sera in different dilutions. In contrast, sera derived from patients with untreated metastatic breast cancer in contrast significantly inhibited the proliferation of control PBMCs in a dilution-dependent manner (serum dilution 1:10, $P < 0.001$; 1:100, $P > 0.1$). Table IV shows the results of two representative experiments out of the performed six assays. Table V shows the successful enrichment by ultracentrifugation of the factor(s) responsible for the suppression of PBMC proliferation in the protein fraction below 25 kDa. This serum preparation resulted in the most effective suppression of PHA stimulation which could not be reversed by dilution (Table V).

**Table III** Correlation of clinical course with changes in PHA-induced stimulation of PBMCs (preoperative values compared with data obtained after 12 months) in patients with stage I–III breast cancer with complete remission after 36 months ($n = 82$)

| *Group* | *Disease status at 36 months* | *Change in mitogenic stimulation*[a] *Increase* | *Decrease* | *Fisher's exact test (two-tailed)* |
|---|---|---|---|---|
| Total | CR | 9 | 58 | <0.0001 |
| | PD | 14 | 1 | |
| Stage | | | | |
| I | CR | 2 | 6 | |
| | PD | – | – | |
| II | CR | 5 | 39 | 0.0027 |
| | PD | 4 | 1 | |
| III | CR | 2 | 13 | <0.0001 |
| | PD | 10 | – | |

Overall sensitivity, 93.30%; overall specificity, 86.70%; positive predictive value, 0.6087; Negative predictive value, 0.9831. [a]Mitogenic stimulation with phytohaemagglutinin.

**Table IV** Influence of sera derived from patients with untreated metastatic breast cancer upon PHA-induced proliferation of PBMCs derived from healthy control individuals

| *Experiment* | *Serum* | *Dilution* | *c.p.m.*[a] |
|---|---|---|---|
| 1 | 0 | | 82.6 ± 10.5 |
| | Control | 1:10 | 86.8 ± 6.1 |
| | | 1:100 | 78.2 ± 4.0 |
| | Patient | 1:10 | 31.6 ± 7.4 |
| | | 1:100 | 85.8 ± 4.5 |
| 2 | 0 | | 102.3 ± 11.8 |
| | Control | 1:10 | 94.7 ± 10.5 |
| | | 1:100 | 85.9 ± 5.7 |
| | Patient | 1:10 | 27.2 ± 7.2 |
| | | 1:100 | 95.0 ± 9.9 |

[a]Counts per minute in PBMC stimulation assays using PHA.

**Table V** Characterisation of the factor(s) present in sera of patients with breast cancer which are responsible for the inhibition of PBMC proliferation

| *Number of experiments* | *Serum fraction* | *Dilution* | *c.p.m.* |
|---|---|---|---|
| 4 | Control sera, unfractionated | 1:10 | 75.8 ± 14.3 |
| | | 1:100 | 82.3 ± 15.9 |
| 6 | Patients' sera, unfractionated | 1:10 | 36.7 ± 8.3 |
| | | 1:100 | 44.7 ± 7.6 |
| 6 | Patients' sera >25 kDa | 1:10 | 35.9 ± 7.0 |
| | | 1:100 | 40.3 ± 9.4 |
| 6 | Patients' sera <25 kDa | 1:10 | 14.3 ± 3.0 |
| | | 1:100 | 10.8 ± 7.4 |

*Correlation of the proliferative status of PBMCs in response to PHA with the clinical status after 3 years*

The data presented in Table III show that, of 59 patients who presented with an increase in the proliferative ability of their PBMCs to PHA when preoperative data and values obtained after 12 months were compared, 58 (98%) remained in complete remission for a total of 3 years. In contrast, of 23 patients with a decrease in the proliferation of their PBMCs in response to PHA within 12 months, only nine (39%; $P<0.0001$) remained in complete remission for 3 years, whereas the remaining 14 patients (61%) developed metastatic disease. As shown in Table III the correlation remains significant when adjusted to disease stage (stage II, $P=0.0027$; stage III, $P<0.001$). These data result in an overall sensitivity of 93.3% and an overall specificity of 86.7%. Adjusting for stage did not alter estimates. Thus, PHA stimulation studied longitudinally constituted an important new parameter.

### Discussion

The present study was performed in order to analyse prospectively and longitudinally the course of PHA-induced stimulation of PBMCs derived from patients with breast cancer during and after adjuvant chemotherapy or endocrine treatment. Moreover, we studied whether the results from assays investigating PHA-induced stimulation of PBMC had a prognostic and predictive value for the further course of breast cancer. In following these aims, blood samples were obtained from each patient before surgery and 6 as well as 12 months thereafter. Independent of the current investigational protocol, patients had been randomised after surgery to receive either adjuvant chemotherapy according to the CMF protocol or tamoxifen or both (EBCTCG, 1992). It was found tht PHA-induced stimulation of PBMCs derived from patients with breast cancer before surgery was significantly lower than in PBMCs from healthy control individuals and correlated with the stage of the disease, including tumour size and lymph node status, i.e. the tumour burden. Although a transient drop in PHA-induced proliferation of PBMCs derived from patients undergoing CMF treatment was noted, 12 months after surgery the mean results of PBMC proliferation assays were similar in all patient groups (i.e. those 6 months after termination of CMF treatment or during tamoxifen therapy or both). However, also at this point of time, data obtained in patients with breast cancer were significantly lower than those from healthy controls, as reported previously by other authors (Whittaker *et al.*, 1971; Stein *et al.*, 1976).

Our data demonstrated, finally that, irrespective of the kind of adjuvant treatment, two-thirds of patients whose PBMCs showed a drop in their PHA-induced proliferation within 1 year experienced rapid recurrence of disease. This fact was further corroborated by the observation that breast cancer did not recur within the observation period in patients whose PBMCs showed an increase in PHA-induced proliferation.

The results presented in the current report pose a series of questions. Thus, the persistent decrease in PHA-induced proliferation of PBMCs derived from patients with breast cancer has to be explained. Considering our data there could be several reasons for this finding. The initial decrease in lymphocyte proliferation could be due to the presence of the tumour and its influence upon the immune system. Such assumptions can be made on the basis of data obtained in humans with cancer as well as in experimental animals which demonstrated decreased DTH and a defect in lymphocyte proliferation in the presence of neoplastic tissue (Stein *et al.*, 1976; Broder *et al.*, 1978; Giuliano *et al.*, 1979). Several reasons have been discussed for these findings, including defective lymphokine production and an increase in the production of suppressor factors by the tumour (Whittaker *et al.*, 1971; Nimberg *et al.*, 1975; North *et al.*, 1984; Mizoguchi *et al.*, 1992).

Previous studies by other investigators (Whittaker *et al.*, 1971; Nimberg *et al.*, 1975; Giuliano *et al.*, 1979; North *et al.*, 1984; Mizoguchi *et al.*, 1992) as well as our data would favour the second possibility, as the exogenous addition of recombinant interleukin 2 to cultures of lymphocytes derived from patients who had presented with defective proliferation previously in our experiments did not have any enhancing influence upon lymphocyte function (data not shown). In contrast, the addition of sera derived from patients with metastatic breast cancer to lymphocytes derived from healthy control individuals produced a significant decrease in PHA-induced proliferation, thus suggesting the presence of one or more suppressive factors of a molecular weight below 25 kDa. Although this explanation could be also considered for the finding of a decrease in lymphocyte proliferation in patients who were to develop metastatic disease within the subsequent 2 years, additional variables, including the induction of suppressor cells by the emerging tumour (Yu *et al.*, 1977; North *et al.*, 1984) or an alteration in signal transduction molecules (Mizoguchi *et al.*, 1992), must be considered. The problem could be further aggravated by the fact that adjuvant cytostatic treatment with CMF has previously been demonstrated in studies in this laboratory to lead to a prolonged defect in the production of a primary immune response following vaccination (Zielinski *et al.*, 1986) and lymphocyte proliferation (Knogler *et al.*, 1992) as well as lymphokine production (Zielinski *et al.*, 1986, 1989*b*). The latter aspects also constitute the most likely explanations for the decrease in PHA-induced lymphocyte proliferation under immediate CMF therapy seen in the present study.

Clinically, the decrease in lymphocyte proliferation in two-thirds of patients who were to develop metastases within the relatively near future clearly constitutes a new prognostic tool for the prediction of the development of early metastatic disease. Nevertheless, owing to the relatively small number of patients and short follow-up times, the ultimate prognostic value of this phenomenon has to be proven in a larger study, which is ongoing in our institute. Although previous studies on a similar question have failed to find such a prognostic value for PHA stimulation (Stein *et al.*, 1976; Nordman *et al.*, 1985) in solid tumours, it is an accepted predictive parameter in Hodgkin's disease (Björkholm *et al.*, 1982; Wedelin *et al.*, 1982). However, the lack of such a finding in patients with solid tumours may be due to the heterogeneity of studied patients so far. Nevertheless, the fact is surprising, as immunological variables have not been used until now as either prognostic tools or for the design of adjuvant trials. However, several studies have demonstrated a clear association of lytic effector lymphocyte function and such established prognostic variables as tumour load (Contesso *et al.*, 1989), oestrogen receptor status (Clark *et al.*, 1988) and amplification of the HER-2neu oncogene (Zeillinger *et al.*, 1989).

Although further studies and the use of more sophisticated stimulants of lymphocyte proliferation than the widely available PHA assay including the use of anti-CD3 antibodies will have to corroborate and extend our results, the results presented in the current report could lead to such clinical consequences as lymphokine treatment or the repeated use of a series of adjuvant cytostatic therapies in expanded time intervals or in selected patients who fulfil the appropriate immunological criteria.

### Abbreviations

DTH, delayed-type hypersensitivity; IL-2, interleukin 2; PBMC, peripheral blood mononuclear cell; PHA, phytohaemagglutinin; CMF, cyclophosphamide, methotrexate, fluoruracil; ER, oestrogen receptor; PgR, progesterone receptor.

### Acknowledgements

The authors thank Dr Susan Hilsenbeck for assistance in statistical analysis and Ms Waclawa Kalinowski, who contributed by providing expert technical assistance.

**References**

BJÖRKHOLM M, WEDELIN C, HOLM G, OGENSTAD S, JOHANSSON B AND MELLSTEDT T. (1982). Immune status of untreated patients with Hodgkin's disease and prognosis. *Cancer Treat. Rep.*, **66**, 701.

BONADONNA G, ROSSI A, VALAGUSSA P, BANFI A AND VERONESI U. (1977). The CMF program for operable breast cancer with positive axillary nodes. *Cancer*, **39**, 2904-2910.

BRODER S AND WALDMANN TA. (1978). The suppressor-cell network in cancer. *N. Engl. J. Med.*, **299**, 1335-1341.

CLARK GM AND MCGUIRE WL. (1988). Steroid receptors and other prognostic factors in primary breast cancer. *Oncology*, **15** (Suppl. 1), 20-25.

CONTESSO G, SACCANIJOTTI G AND BONADONNA G. (1989). Tumor grade as prognostic factor in primary breast cancer. *Eur. J. Cancer Clin. Oncol.*, **25**, 403-9.

CUNNINGHAM-RUNDLES S, FILLIPA DA, BRAUN DW, ANTONELLI P AND ASHIKARI H. (1981). Natural cytotoxicity of peripheral blood lymphocytes and regional lymph node cells in breast cancer in women. *J. Natl Cancer Inst.*, **67**, 585-590.

EARLY BREAST CANCER TRIALISTS COLLABORATIVE GROUP (EBCTCG). (1992). Systemic treatment of early breast cancer by hormonal, cytotoxic, or immune therapy: 133 randomised trials involving 31,000 recurrences and 24,000 deaths among 75,000 women. *Lancet*, **i**, 1-15, 71-85.

FEARON ER, PARDOLL DM, ITAYA T, GOLUMBEK P, LEVITSKY HI, SIMONS JW, KARASUYAMA H, VOGELSTEIN B AND FROST B. (1990). Interleukin-2 production by tumor cell bypasses T helper function in the generation of an antitumor response. *Cell*, **60**, 397-403.

GIULIANO AE, RANGEL D, GOLUB SH, HOLMES CE AND MORTON DL. (1979). Serum-mediated immunosuppression in lung cancer. *Cancer*, **43**, 917-924.

KIRCHENER H. (1978). Suppressor cells of immune reactivity in malignancy. *Eur. J. Cancer*, **14**, 453-459.

KNOGLER W, KUBISTA E AND ZIELINSKI CC. (1992). Prolonged decrease in mitogenic stimulation of peripheral blood mononuclear cells following adjuvant chemotherapy, but not under tamoxifen, in stage II breast cancer. *Cancer J.*, **5**, 32-3.

MIKI J, AIGINGER P, CZERWENKA K, KUBISTA E, SALZER H, SEVELDA P, SPONA J, STAFFEN A AND ZIELINSKI CC. (1990). Adjuvant tamoxifen in postmenopausal stage II breast cancer five years on. *Lancet*, **335**, 541-542.

MIZOGUCHI H, O'SHEA JJ, LONGO DL, LOEFFLER CM, MCVICAR DW AND OCHOA AC. (1992). Alterations in signal transduction molecules in T lymphocytes from tumor-bearing mice. *Science*, **258**, 1795-1798.

NIMBERG RB, GLASGOW AH, MENZOIAN JO, CONSTANTIAN MB, COOPERBAND SR, MANNICK JA AND SCHMID K. (1975). Isolation of an immunosuppressive peptide fraction from the serum of cancer patients. *Cancer Res.*, **35**, 1489-1494.

NORDMAN E, LEHTO I AND TOIVANEN A. (1985). Immune functions and the prognosis of patients with solid tumours. *Cancer Immunol. Immunother.*, **20**, 38-42.

NORTH RJ AND BURSUKER I. (1984). Generation and decay of the immune response to a progressive fibrosarcoma. *J. Exp. Med.*, **159**, 1295-1311.

PENN I. (1982). The occurrence of cancer in immune deficiencies. *Curr. Prob. Cancer*, **6**(10), 1-64.

PENN I. (1988). Cancer is long-term hazard of immunosuppressive therapy. *J. Autoimmunity*, **1**, 545-548.

STEIN JA, ADLER A, BEN EFRAIM S AND MAOR M. (1976). Immunocompetence, immunosuppression, and human breast cancer. *Cancer*, **38**, 1171-1187.

TICHATSCHEK E, ZIELINSKI CD, MÜLLER CH, SEVELDA P, KUBISTA E, CZERWENKA K, SPONA J, WOLF H AND EIBL MM. (1988). Long-term influence of adjuvant therapeutic measures upon natural killer cell activity in breast cancer. *Cancer Immunol. Immunother.*, **27**, 278-283.

UCHIDA A AND HOSHINO T. (1980). Clinical studies on cell-mediated immunity in patients with malignant disease. *Cancer Immunol. Immunother.*, **9**, 153-158.

VOSE BM AND MOORE M. (1980). Heterogeneity of suppressor of mitogen responsiveness in human malignancy. *Cancer Immunol. Immunother.*, **9**, 163-172.

WEDELIN C, BJÖRKHOLM M, HOLM G, OGENSTAD S, JOHANSSON B AND MELLSTEDT H. (1982). Lymphocyte function in untreated Hodgkin's disease: an important predictor of prognosis. *Br. J. Cancer*, **45**, 70.

WHITE D, JONES DB, COOKE T AND KIRKHAM N. (1982). Natural killer (NK) activity in peripheral blood lymphocytes of patients with benign and malignant breast disease. *Br. J. Cancer*, **46**, 611-6.

WHITTAKER MG, REES K AND CLARK CG. (1971). Reduced lymphocyte transformation in breast cancer. *Lancet*, 892-893.

YU A, WATTS H, JAFFE N AND PARKMAN R. (1977). Concomitant presence of tumor-specific cytotoxic and inhibitor lymphocytes in patients with osteogenic sarcoma. *N. Engl. J. Med.*, **297**, 121-127.

ZIELINSKI CC, STULLER I, DORNER F, MÜLLER C AND EIBL M. (1986). Impaired primary but not secondary immune response in patients with breast cancer under aduvant therapy. *Cancer*, **58**, 1648.

ZEILLINGER R, KURY F, CZERWENKA K, KUBISTA E, SLIUTZ G, KNOGLER W, HUBER J, ZIELINSKI C, REINER G, JAKESZ R, STAFFEN A, REINER A, WRBA F AND SPONA J. (1989). HER-2 amplification, steroid receptors and epidermal growth factor receptor in primary breast cancer. *Oncogene*, **4**, 109-112.

ZIELINSKI CC, TICHATSCHEK E, MÜLLER C, KALINOWSKA W, SEVELDA P, CZERWENKA K, KUBISTA E AND SPONA J. (1989*a*). Association of increased lytic effector cell function with high estrogen receptor levels in tumour-bearing patients with breast cancer. *Cancer*, **63**, 1985-1989.

ZIELINSKI CC, MÜLLER C, TICHATSCHEK E AND AIGINGER P. (1989*b*). Decreased production of soluble interleukin 2 receptor by phytohaemagglutinin-stimulated peripheral mononuclear cells in patients with breast cancer after adjuvant therapy. *Br. J. Cancer*, **60**, 712-714.